# Prenatal Exophthalmia Revealing a Postnatal Orbital Teratoma

**DOI:** 10.1155/2020/1597353

**Published:** 2020-07-13

**Authors:** Ahgbatouhabeba Ahnoux-Zabsonre, Jérôme Sanou, Yérénou Ferdinand Lankoandé, Chantal Bouda, Gertrude Méda, Assita Lamien-Sanou

**Affiliations:** ^1^UFR/SDS University Joseph Ki Zerbo, 03 BP7021, Ouagadougou, Burkina Faso; ^2^Yalgado Ouedraogo University Hospital Center, Department of Ophthalmology, 03 BP7022, Captain Sankara Avenue, Ouagadougou, Burkina Faso; ^3^Yalgado Ouedraogo University Hospital Center, Department of Paediatrics, 03 BP7022, Captain Sankara Avenue, Ouagadougou, Burkina Faso; ^4^Yalgado Ouedraogo University Hospital Center, Department of Pathological Anatomy, 03 BP7022, Captain Sankara Avenue, Ouagadougou, Burkina Faso

## Abstract

**Purpose:**

Teratomas are congenital tumors of stem cells derived from the three germ layers. They are frequently located in the sacrococcygeal region. Orbital teratoma is rare with less than 70 cases reported until 2016. We report the case of prenatal exophthalmia discovered by ultrasound exam which turned out to be a teratoma postnatally. The newborn in our case was female, just as described in the literature. Treatment consisted of total removal of the teratoma and the eyeball. Reconstructive surgery remains a big challenge since our medical technology is limited.

**Conclusion:**

A prenatal exophthalmia on fetal ultrasound should make us think of a teratoma, even if it is very rare. Fetal orbital teratoma may be associated with fetal survival. The infant will benefit from a reconstructive surgery of the orbit.

## 1. Introduction

Teratomas are congenital stem cell tumors derived from the three germ layers. Histological exam classifies these tumors as mature or well-differentiated teratoma and immature teratoma, depending on the degree of cell differentiation [1]. Immature teratoma has the tendency to turn into a malignant tumor [1]. Teratoma is twofold prevalent in women as compared to men. The most frequent localization of such tumor is the sacrococcygeal region [1, 2]. Teratoma is rarely localized in the orbits [2, 3]. It can appear in the orbit primitively or secondarily from the spread of intracranial teratomas [4, 5]. Teratoma is treated surgically by the exeresis of the tumor while sparing the ocular globe [6]. We report a case of antenatal exophthalmia that appeared to be a teratoma postnatally.

## 2. Case Report

**Figure 1:**
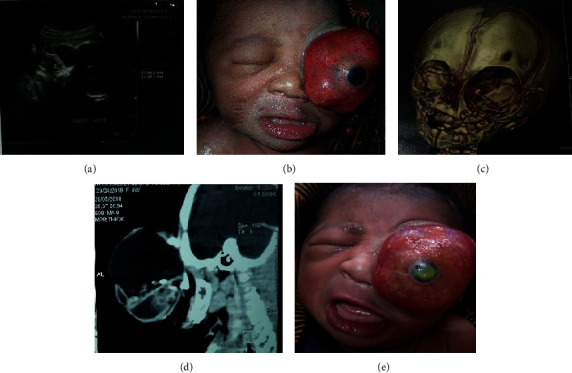
(a) Transabdominal ultrasound of the mother at 36 weeks of pregnancy showing a right orbital mass. (b) Congenital exophthalmia (5 cm). (c) CT scan of the head and the orbits: heterogeneous mass in the left orbit with cystic, calcified, fatty, and vascularized tissues. (d) 3D skull scan: enlarged left orbital cavity. (e) Left eye: corneal abscess with perforation.

We report the case of a newborn who was referred to the ophthalmologic clinic of Yalgado Ouédraogo University Hospital for appropriate care of a left side congenital exophthalmia that was discovered prenatally through a routine transabdominal ultrasound of the mother (Figure 1(a)). An MRI on the mother was prescribed prenatally but could not be realized due to shortage of money.

The newborn was seen on the first day of life and was a female baby who had a term vaginal delivery with an APGAR score of 9/9/10, a weight of 2,700 g, and a head circumference of 31 cm. No pathological family history was recorded. There was no consanguinity between the 19-year-old primipara mother and the 30-year-old father. Ophthalmic exam was normal for the right eye while showing on the left eye (Figure 1(b)) a multilobulated hypervascularized, nonpulsatile, nonreducible massive proptosis. The cornea and the iris were normal. The anterior chamber was narrow, and the pupil was small. A thorough physical exam performed by a pediatrician did not reveal other abnormalities.

Abdominal and pelvic ultrasounds were normal. Blood cell count and biochemistry tests were normal. Computerized tomography scan of the head and orbits revealed a heterogeneous mass with cystic, calcified, fatty, and vascularized tissues (Figure 1(c)). The orbital cavity was enlarged with no bone lyses (Figure 1(d)).

Despite antibiotic treatment along with eye lubricants, the newborn developed, on his 6th day of life, corneal abscess and corneal perforation (Figure 1(e)).

**Figure 2:**
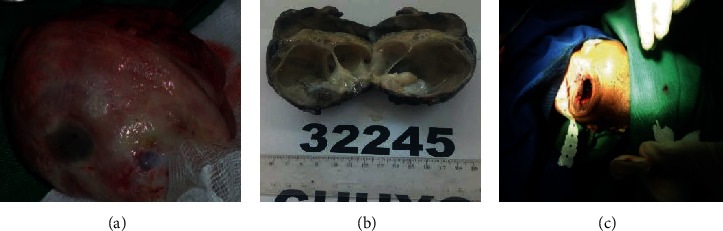
(a) Tumor removed. (b) Empty orbital cavity after mass exeresis. (c) Gross appearance of the mass : a multilocular cyst.

Surgical treatment consisted in removing the entire mass (Figure 2(a)) while sparing the eyelids. The orbital cavity remained empty (Figure 2(b)).

**Figure 3:**
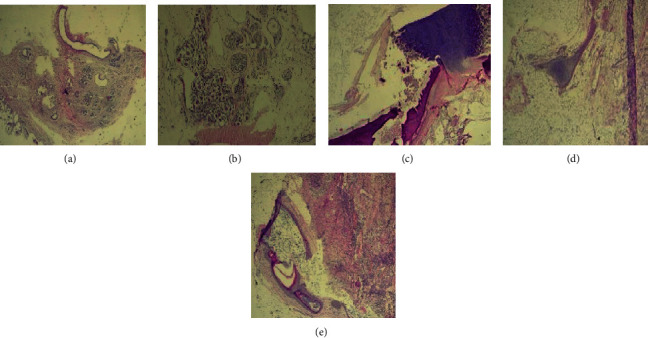
(a) Histological examination showing respiratory tissue. (b) Histological examination showing a cluster of neuroganglion cells and nerve cells. (c) Histological examination showing cartilage tissue (top left) and bone tissue (bottom). (d) Histological examination showing a cartilaginous lobule in the middle of an adipose lobule. (e) Histological examination showing keratinized malpighian epithelium.

Gross appearance of the mass (Figure 2(c)) revealed a multilocular cyst with clear substance and areas without endocystic vegetations. Microscopic exam showed that the cyst was boarded by a layer of mature tissues such as respiratory (Figure 3(a)) or intestinal (Figure 3(b)) type linings, keratinized malpighian epithelium (Figure 3(c)), cartilaginous nodules (Figure 3(d)), clusters of lymphoid cells, and nervous tissues (Figure 3(e)). There were no findings that were suspicious of malignancy. All findings rather pointed to a multitissular mature cystic teratoma of the left eye.

**Figure 4:**
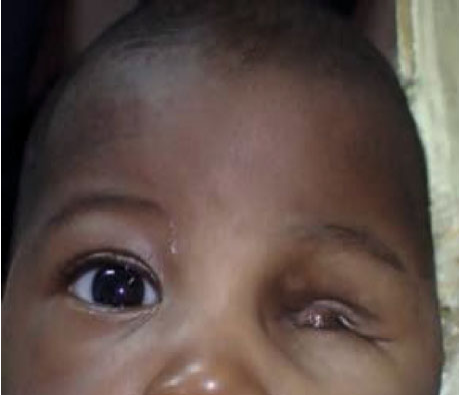
Left orbital cavity to be repaired.

Postsurgery outcomes were without any complications (Figure 4). The remaining challenge was the reconstruction of the orbital cavity since we did not have the means of performing such a surgery.

## 3. Discussion

The first orbital tumor compatible with teratoma was described by Holmes in 1863 [7]. Orbital teratoma is an extremely rare tumor developed from primitive germ cells [8]. In fact, Pellerano et al. reported only 70 cases of such tumor throughout English literature until 2016 [9]. The present report is the first documented case of the disease in Burkina Faso.

Orbital teratoma can be characterized at birth by a massive unilateral exophthalmia [10–12]. Our report has the particularity of showing an exophthalmia that was noticed antenatally through ultrasound exam, so its prenatal diagnosis has been made. Such findings have prompted the prescription of an MRI on the mother but the MRI could not be run due to money shortage. Prenatal diagnosis of teratoma with voluminous exophthalmia has rarely been documented. We were able to find three documented cases. The first one had led to therapeutic abortion. Histological exam had confirmed the diagnosis of orbital teratoma [13]. The second case described a newborn who benefited from an exenteration and an orbital prosthesis [3]. The third case reported a suspicion of orbital teratoma through prenatal MRI. However, the teratoma was ruptured and led to in utero fetal death [14]. The present case report is therefore the second case in which the child was born with the tumor. However, we were not able to spare the eyeball in our treatment. The newborn of our case report was female; this corroborates with findings in other studies [2].

Massive exophthalmia may lead to exposure keratopathy and its complication such as abscess formation and eye perforation [9, 11, 12, 15]; this is what happened with our patient.

Postnatal CT scan of the brain, just like in our case report, usually shows an enlarged orbit with no sign of bone destruction in which can be found a heterogeneous mass containing vascularized, fatty, or calcified tissues and orbital cysts [11].

Histological exam of the removed tumor of our patient revealed various tissues (gastrointestinal, cerebral, or cartilaginous and fatty tissues) which were mature. These mature features usually rule out the risk of the tumor turning malignant [1, 16] as opposed to immature teratoma that has a high risk of malignancy [1, 16].

Common surgical treatment is the exeresis of the tumor while sparing the eyeball whenever possible [6, 17–21]. However, exenteration often appears to be the only therapeutic option [3, 11, 22]. This happened with our patient who had a prenatal massive exophthalmia with early corneal complications. Orbital reconstructive surgery is required in advance cases for esthetic purpose. This could not be recommended to the parents of our patients due to lack of adequate surgical means. Medical evacuation was therefore recommended [11, 19, 23].

Orbital teratoma is an extremely rare congenital pathology; it can be discovered prenatally using ultrasounds. Thereafter, MRI can point to this pathology when showing exophthalmia and allows early treatment of the newborn while sparing the eyeball whenever possible. Otherwise, surgery with mutilations is required with challenges of performing eyeball reconstruction on a growing child.

## Data Availability

The data used to support the findings of this study are available from the corresponding author upon request.
